# Providing a model for financing the treatment costs during biological crises using the fiscal space development approach

**DOI:** 10.1186/s13561-023-00450-x

**Published:** 2023-08-01

**Authors:** Maryam Yaghoubi, Masoud Vahedi Idehlo, Parisa mehdizadeh, Mohammad Meskarpour Amiri

**Affiliations:** 1 Health Management Research Center, Baqiyatallah University of Medical Sciences, Tehran, Iran; 2 Independent Researcher, Tehran, Iran

**Keywords:** Financial Management, Healthcare Financing, Pandemics

## Abstract

**Background:**

Expanding fiscal space for health can be defined as providing additional budgetary resources for health, which is highly important during biological crises. This study aimed to provide a model for financing the treatment costs during biological crises using the development of the fiscal space approach.

**Methods:**

This study employed a descriptive mixed-method design, consisting of three stages. In the first stage, a systematic review of relevant literature was conducted using multiple databases, including Scopus, PubMed, and Google Scholar. A total of 45 studies that met the inclusion criteria were selected. In the second stage, a panel of 14 experts identified five primary and 32 secondary strategies using an open questionnaire. Any additional strategies not identified during the literature review were added if a consensus was reached by experts. In the final stage, the Best Worst Method (BWM) was used to prioritize the identified strategies and sub-strategies based on their feasibility, effectiveness, quick yield, and fairness.

**Results:**

Five strategies and fifty sub-strategies were identified. The most important strategies were the increase in health sector-specific resources (0.3889), increase in efficiency of health expenditures (0.2778), structural reforms (0.1111), health sector-specific grants and foreign aid (0.1667), and conducive macroeconomic conditions (0.05556). The most important sub-strategies were establishing and increasing earmarked taxes for the health sector (0.0140), expanding Universal Health Coverage (UHC) plans (0.0103), attracting the participation of non-governmental organizations (NGOs) and charitable organizations in the health sector (0.0096), integrating basic social insurance funds (0.0934), and tax exemptions for economic activists in the health sector (0.009303) during the crisis.

**Conclusion:**

This study identified five main strategies and 50 sub-strategies for financing the treatment costs during biological crises. The most important strategies were increasing health sector-specific resources, improving efficiency of health expenditures, and implementing structural reforms. To finance health expenditures, harmful and luxury goods taxes can be increased and allocated to the health sector during crises. UHC plans should be improved and expanded, and the capacity of NGOs and charitable organizations should be better utilized during crises.

**Supplementary Information:**

The online version contains supplementary material available at 10.1186/s13561-023-00450-x.

## Introduction

Universal Health Coverage (UHC) is a key goal for policymakers worldwide, aimed at ensuring that all people have access to adequate healthcare without facing financial hardship. However, achieving UHC is challenging, especially given the high cost of medical treatment, particularly for those with low incomes [1]. Over the years, healthcare costs have continued to rise globally, driven by several factors such as social and epidemiological transitions and changes in health behaviors that are beyond the control of health systems. Nevertheless, certain controllable factors, such as the expansion of access to services, increased variety, and cost of healthcare services, the adoption of new and expensive technologies, and reimbursement mechanisms for healthcare providers, also contribute to rising healthcare costs [2, 3]. To achieve UHC, policymakers must explore innovative sterategies to reduce healthcare costs and manage these controllable factors effectively, making healthcare accessible and affordable for all.

The COVID-19 pandemic has highlighted the vulnerability of health systems and the critical need for Universal Health Coverage (UHC) during biological crises. The COVID-19 pandemic has had a devastating impact on the global economy and has put immense pressure on developing countries’ budgets, particularly for health expenditures. This has had a significant impact on efforts towards achieving UHC [4]. With the risk of future biological crises, it is crucial to conduct further research and investigate how such crises can be adequately funded, especially given the limited financial resources available in the health system. The rapid spread of biological factors can lead to national and transnational crises, making it essential to prepare adequately to deal with them. Given the substantial costs associated with responding to these incidents, it is vital to develop robust plans for funding. As policymakers, we must explore innovative financing mechanisms and collaborations between public and private sectors to ensure adequate and sustainable funding to respond to future biological crises.

Adequate and stable financing for medical expenditure is crucial not only in developing countries but also in developed ones. The COVID-19 pandemic has highlighted the importance of such financing. For instance, a study in the United States estimated that the cost of a single symptomatic COVID-19 case during the infection period was $3045. If 20% of the US population were to get infected, it is projected that there could be a median of 11.2 million hospitalizations, 2.7 million ICU admissions, 1.6 million patients requiring a ventilator, 62.3 million hospital bed days, and $163.4 billion in direct medical costs during the course of the pandemic [5]. This figure underscore the need for robust and sustainable financing mechanisms to ensure that healthcare systems are adequately prepared to respond to public health emergencies such as the COVID-19 pandemic.

The notion of fiscal space was first introduced after the Asian crisis to emphasize the importance of maintaining funding for basic infrastructure, even during periods of fiscal consolidation. Fiscal space refers to the availability of financial resources within the government’s budget, which can be allocated towards specific priorities without compromising the stability of the economy or the government’s financial position. The development of fiscal space is crucial for countries to meet the increasing demand for financial stability and to uphold their political commitments to UHC. Ultimately, the development of fiscal space is a critical component in ensuring that healthcare systems have adequate and sustainable financing to meet the needs of their populations [6].

Developing countries have had to re-evaluate their fiscal space for health financing due to the rise in health costs in the wake of the aging population and financial crises. The recent Covid-19 pandemic has further highlighted the urgency of this issue. For instance, Jahanmehr et al. suggest that developing fiscal space in Iran could be achieved by improving the efficiency of existing health expenditures, and there is potential to earmark resources to strengthen Iran’s health system based on expert opinions [7]. Also, During the Covid-19 crisis, Pakistan utilized the increased dedicated resources to the health sector (i.e., the allocated budget), grants and foreign aid for the health sector, and the efficiency of health expenditures [8]. In Ghana its suggested that to fund health expenditures, the government should raise taxes and improve tax collection [9]. Kutzin and Sparkes highlight the importance of health system strengthening, universal health coverage, health security, and resilience to increase fiscal space in the health sector of developing countries by increasing the share of the health sector in GDP and creating support funds to cover healthcare costs [10].

The COVID-19 pandemic has brought to light the significant financial challenges that can arise in healthcare systems due to the rapid spread of biological agents. Thus, it is essential to establish the necessary preparedness by developing the fiscal space of the health sector to tackle such crises. Given the destructive impacts of biological crises on the healthcare sector, providing for the associated costs has become increasingly crucial. However, the scarcity of financial resources and rising treatment costs necessitate exploring various cases and devising an effective model for financing treatment costs during biological crises. To the best of our knowledge, no study has yet provided a model for financing treatment costs during biological crises using a fiscal space development approach. Therefore, the present study aims to fill this gap by developing a model for financing treatment costs during biological crises using the fiscal space development approach, which can improve the financial preparedness of the healthcare system and enhance its overall performance during such crises.

## Methods

### Study design

This study, conducted in 2021, used a mixed-methods approach to identify effective strategies for the development of fiscal space for the healthcare sector during biological crises. The study included two main components: a systematic review and an expert panel. The systematic review was conducted according to the PRISMA guidelines and aimed to identify strategies that have been previously proposed and tested in the literature. On the other hand, the expert panel gathered expert opinions to identify latent strategies that may not have been explicitly discussed in the literature. Furthermore, the study included a prioritization step in which sub-strategies identified from the literature review and expert opinions were evaluated and ranked based on their importance. This was done using the Best-Worst Method (BWM), which allowed the panel of experts to identify the most important strategies for developing the fiscal space of the health sector during biological crises.

### Data sources

The first stage of the study involved a systematic review of the literature, which was conducted by searching several databases including Scopus, PubMed, and Google Scholar for eligible studies published from January 2003 to August 2022. The search terms or keywords used included Health, Fiscal Space, Financing, Resource Mobilization, Domestic Resource, etc. The complete search strategy and the number of articles found in each database are presented in the appendix A.

In the second stage of the study, 14 experts were engaged in a detailed discussion of the strategies and sub-strategies extracted from the systematic review using an open questionnaire. They were asked to identify the necessary criteria for developing fiscal space in biological crises. In addition, experts identified additional strategies that were not identified in the literature review in a separate box for each domain, which were added to the list if consensus among the experts was reached. Table 1 presents the demographic characteristics of the experts involved in examining the strategies and sub-strategies.

**Table 1:** Demographic characteristics of experts in the stage of examining the strategies and sub-strategies

Variables	Modes	N (%)
**Gender**	**Male**	(66.66)6
	**Female**	(33.33)3
**Education status**	**Master**	(22.22)2
	**Ph.D. and above**	(77.77)7
**Field of study**	**Health economic**	(33.33)3
	**Healthcare management**	(66.66)6
**Type of employment**	**Faculty member**	(22.22)2
	**Staff member**	(77.77)7

In the third stage, the identified strategies and sub-strategies in the first and second stages were prioritized by ten experts and using the BWM. Table 2 shows the demographic characteristics of experts in the stage of prioritizing the strategies and sub-strategies.

**Table 2:** Demographic characteristics of experts in the stage of prioritizing strategies or sub-strategies

Variables	Modes	N (%)
**Gender**	**Male**	(60.00)6
	**Female**	(40.00)4
**Education status**	**Master**	(20.00)2
	**Ph.D. and above**	(80.00)8
**Field of study**	**Health economic**	(30.00)3
	**Healthcare management**	(70.00)7
**Type of employment**	**Faculty member**	(40.00)4
	**Staff member**	(60.00)6

### Inclusion and exclusion criteria

For the systematic review, inclusion criteria were set to identify relevant studies published in English or Persian language between January 2003 and August 2022 that focused on financing healthcare expenditures, fiscal space development of the health system, and financing during biological crises. Studies that did not meet these criteria or had incomplete full-text articles were excluded.

To be eligible for participation in the study, experts were required to have at least 5 years relevant work experience, research experience, or academic credentials in the field of financing healthcare expenses, as well as familiarity with the field of biological threats and incidents. Conversely, study participants were excluded if they expressed a lack of willingness to cooperate or allocate sufficient time to complete the questionnaire, completed an incomplete questionnaire, or lacked valid experience in the field of financing healthcare expenses.

### Data collection procedures

**Fig. 1:**
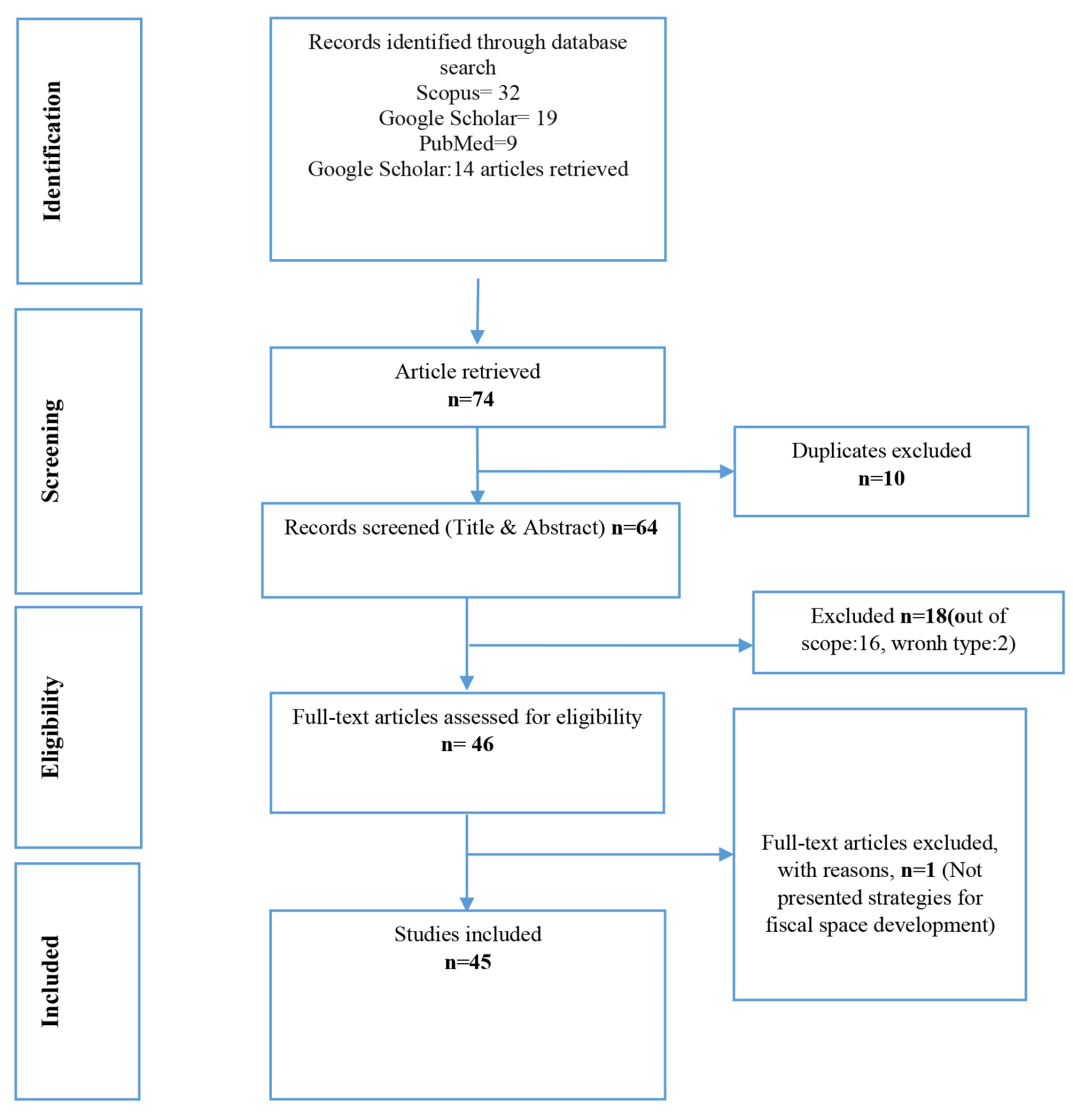
PRISMA flow chart diagram of the systematic reviwe

At the systematic review, the records obtained from the databases were initially screened for eligibility by two reviewers (MV and MMA) and duplicates were removed using EndNote software. Thereafter, the remaining records were screened independently by two reviewers (PM and MY) based on titles and abstracts. In case of disagreement, a third independent researcher (MMA) was consulted for resolution. The review process is depicted in the PRISMA flow diagram (Fig. 1), which shows the number of studies identified, screened, assessed for eligibility, and included in the systematic review, along with the reasons for exclusion.

In order to data extraction we followed the methodology described in Nayak et al. to further enhance the methodology and provide better insights into the data extraction process [11]. a table was created for categorizing and extracting data from the articles, which included the main information of the articles. This information included the year of publication, the studied country, the article author, the relevant solution, and the related sub-solution of the article. Then, the full text of all articles was carefully studied and the above information was extracted and analyzed narratively. In order to ensure the quality of the extracted information and the analyzed results, triangulation was used, in a way that sterategies and sub-sterategies were separately extracted by two researchers, and then all extracted sterategies were reviewed and standardized in a group discussion session by the research team members (4 people). The actions in this stage included removing duplicate sub-sterategies, merging identical sub-sterategies expressed in different literature in the researches, and matching sub-sterategies with main sterategies to prevent wrong connections between sterategies and sub-sterategies. Finally, a table was prepared for the second phase of the study, which included the main solution, sub-solution, number of repetitions, and relevant references.

### Data analysis methods

The study included a prioritization step in which sub-strategies identified from the literature review and expert opinions were evaluated and ranked based on their feasibility, effectiveness, quick yield, and fairness. This was done using the Best-Worst Method (BWM) at Lingo software, which allowed the panel of experts to identify the most important strategies for developing the fiscal space of the health sector during biological crises. The five data analysis phases of BWM were as follows:

Step 1) Determine a set of decision criteria: In this step, the decision maker considers the criteria (12cn) that should be used to arrive at a decision.

Step 2) Determine the best (e.g., the most desirable, most important) and worst (e.g., the least important, most unpleasant) criteria. In this step, the decision maker determines the best and worst criteria. No comparison is made at this stage.

Step 3) Determine the preference of the best criterion over all the other criteria using a number between 1 and 9. The resulting Best-to-Others (BO) vector would be:

BO(B,j) = w(B)/w(j).

Where BO(B,j) is the preference of the best criterion B over criterion j, w(B) is the weight of the best criterion B, and w(j) is the weight of criterion j.

Step 4) Determine the preference of all the criteria over the worst criterion using a number between 1 and 9. The resulting Others-to-Worst (OW) vector would be:

OW(j,W) = w(j)/w(W).

Where OW(j,W) is the preference of criterion j over the worst criterion W, w(j) is the weight of criterion j, and w(W) is the weight of the worst criterion W.

Step 5) Find the optimal weights (w1*,w2*,…,wn*):

The optimal weights for the criteria are the ones where, for each pair of B and j, we have:

BO(B,j) = w(B)/w(j) > = OW(j,W) = w(j)/w(W).

To satisfy these conditions for all j, we should find a solution where the maximum absolute differences |BO(B,j) - OW(j,W)| for all j are minimized. This can be formulated as a linear programming problem as follows:

minimize: max{|BO(B,j) - OW(j,W)|}

subject to:

sum(wi) = 1 (the weights sum to 1).

wi > = 0 (the weights are non-negative).

Once this problem is solved, the optimal weights (w1*,w2*,…,wn*) are obtained. Using the value of the objective function, we can calculate the consistency ratio to assess the consistency of the decision maker’s preferences.

### Ethical considerations and approval

This study was conducted in compliance with all the principles of professional and scientific ethics. The study protocols were approved by Iran National Committee for Ethics in Biomedical Research with the code no. IR.BMSU.REC.1399.427.

## Results

Table 3 shows the identified strategies and sub-strategies based on the frequency, reference numbers, and whether were identified by experts or extracted from the studies. In our study, five strategies (structural reforms, increase in efficiency of health expenditures, health sector-specific grants and foreign aid, conducive macroeconomic conditions, and increase in health sector-specific resources) and 50 sub-strategies were identified.

**Table 3:** Strategies and sub-strategies identified for the development of the fiscal space in the field of health

Strategies	Sub-strategies	Fsr	Citation	Experts
**Structural reforms**	Precedence of prevention over treatment in health budgetary allocation	11	[8, 12, 14, 18, 28, 33, 34, 41–44]	
	Reprioritization of the health budget within the government budget	10	[15–18, 25, 44–48]	
	Budget flexibility in special circumstances	6	[15, 16, 18, 30, 35, 49]	
	Budget prioritization for health based on changes in epidemiology and disease burden	5	[16–18, 35, 50]	
	Modeling and forecasting the growth trend of health costs and financial strategies	5	[16, 17, 18, 35, 50]	
	Increasing the share of insurance (private and public) and prepaid financial resources in health system financing	3	[29, 30, 51]	
	Establishing R&D centers and applied research in the field of health system financing	2	[15, 52]	
	Development of zero-based budgeting	1	[12]	
	Budgetary allocation based on achievements in the treatment sector	1	[53]	
	Allocating a certain percentage of the country’s GDP to the health sector			$\checkmark$
	Establishment and development of an operational budgeting approach (based on performance) in the health system			$\checkmark$
	Allocating part of the budget to encourage achievements related to health			$\checkmark$
**Increase in Efficiency of Health Expenditures**	Evaluating the optimal use of available resources and reducing waste of resources	17	[9, 12, 14, 16–18, 29, 34, 41, 42, 44, 49, 53–55]	
	Development of universal health coverage plans	12	[9, 12, 16, 17, 28, 30, 34, 41, 42, 52, 53, 55]	
	Increasing skill development (or skill enhancement) of healthcare workers	10	[9, 14, 17, 26, 33, 41, 42, 49, 55, 56]	
	Evaluating the efficiency of budgetary allocation in the health sector	8	[12, 17, 26, 33, 41, 44, 52, 56]	
	Supply and demand management in the health sector and supply chain improvement and strategic purchase of drugs and equipment	7	[12, 14, 18, 44, 53, 56]	
	Prevention of corruption, rent, and conflict of interest in health governance	7	[14, 18, 25, 41, 42, 49, 57]	
	Development of public health education, self-care, and the level of literacy and health capabilities of society	6	[12, 17, 18, 33, 37, 56]	
	Modifying the payment mechanism to health service providers	5	[17, 18, 30, 46, 58]	
	Use of decision support systems and evidence-based policy development	4	[9, 16, 52, 59]	
	Multi-layered financing of the health system to allocate public resources to vulnerable groups			$\checkmark$
	Development of resistance economy approach in the health system			$\checkmark$
	Integrating basic social insurance funds			$\checkmark$
	Outsourcing services with an emphasis on systematic monitoring			$\checkmark$
	Strengthening the monitoring of the performance of service delivery units			$\checkmark$
	Identifying and reducing induced demand in providing health care based on clinical guidelines			$\checkmark$
	Identifying and reducing the moral risks of the insured in the health system			$\checkmark$
	Separation of the roles of supervision, provision of care, and monitor the health system			$\checkmark$
**Health Sector-Specific Grants and Foreign Aid**	Use of foreign aid	10	[12, 16, 18, 25, 26, 30, 35, 46, 49, 56]	
	Foreign borrowing	9	[12, 17, 26, 30, 33–35, 56, 57]	
	Foreign aid through technology transfer	2	[16, 56]	
**Conducive Macroeconomic Conditions**	Development of the country’s taxation system	11	[12, 30, 35, 37, 41, 46, 49, 52, 54, 55]	
	Planning to reduce sanctions and the economic effects it caused	3	[7, 16, 18]	
	Increasing the government budget balance	2	[7, 60]	
	Reducing economic dependence on oil exports and underground resources	2	[7, 18]	
	Control and management of the health sector inflation			$\checkmark$
**Increase in Health Sector-Specific Resources**	Establishing and increasing earmarked taxes for the health sector	19	[7, 15, 17, 28, 30, 33–35, 44, 45, 46, 48, 49, 52, 54, 57, 61–63]	
	Encouraging private investment in the health sector	9	[12, 34, 41, 44–46, 49, 51, 64]	
	Attracting the participation of non-governmental organizations and philanthropists in the health sector	4	[25, 35, 62, 64]	
	Allocation of resources from the targeting of subsidies to the health sector	3	[16, 18, 47]	
	Withdrawal from financial funds and strategic reserves of the country in times of health crisis			$\checkmark$
	Development of joint healthcare plans and investments with other government agencies			$\checkmark$
	donation-based crowdfunding and voluntary participation of people			$\checkmark$
	Encouraging foreign investment in health			$\checkmark$
	Using the capacity of the capital market for macro healthcare project financing			$\checkmark$
	Tax exemptions for economic activists in the health sector during the crisis			$\checkmark$
	Allocating a share of public service payments to the health sector			$\checkmark$

As shown in Table 3, the most common sub-strategy related to structural reforms was the re-prioritization of the health budget within the government budget. The most common sub-strategy related to the increase in efficiency of health expenditures was evaluating the optimal use of available resources and reducing the waste of resources. Also, the most common sub-strategy for health sector-specific grants and foreign aid and conducive macroeconomic conditions was, establishing and increasing earmarked taxes for the health sector, using foreign aid, and developing the country’s taxation system, respectively.

Table 4 represents strategies and sub-strategies based on the four criteria: feasibility, effectiveness, quick yield, and fairness using BWM. The most important strategies identified for fiscal space development for health during biological crises were the establishing and increasing earmarked taxes for the health sector (0.0140), expanding UHC plans (0.0103), attracting the participation of NGOs and charitable organizations in the health sector (0.0096), integrating basic social insurance funds (0.00934), and tax exemptions for economic activists in the health sector (0.009303) during the crisis.

**Table 4:** Prioritizing sub-strategies by experts

Strategy	Sub-strategy	Weight	Rank
**Structural reforms**	Reprioritization of the health budget within the government budget	0.00403	27
	Precedence of prevention over treatment in health budgetary allocation	0.00216	38
	Budget flexibility in special circumstances	0.00215	39
	Budget prioritization for health based on changes in epidemiology and disease burden	0.00213	40
	Modeling and forecasting the growth trend of health costs and financial strategies	0.00184	42
	Increasing the share of insurance (private and public) and prepaid financial resources in health system financing	0.00172	43
	Establishing R&D centers and applied research in the field of health system financing	0.00169	44
	Development of zero-based budgeting	0.00148	46
	Budgetary allocation based on achievements in the treatment sector	0.00304	33
	Allocating a certain percentage of the country’s GDP to the health sector	0.00292	34
	Establishment and development of an operational budgeting approach (based on performance) in the health system	0.00282	35
	Allocating part of the budget to encourage achievements related to health	0.00249	36
**Increase in Efficiency of Health Expenditures**	Evaluating the optimal use of available resources and reducing waste of resources	0.00524	17
	Development of universal health coverage plans	0.01036	2
	Increasing skill development (or skill enhancement) of healthcare workers	0.00471	23
	Evaluating the efficiency of budgetary allocation in the health sector	0.00547	15
	Supply and demand management in the health sector and supply chain improvement and strategic purchase of drugs and equipment	0.00458	24
	Prevention of corruption, rent, and conflict of interest in health governance	0.00539	16
	Development of public health education, self-care, and the level of literacy and health capabilities of society	0.00488	21
	Modifying the payment mechanism to health service providers	0.00552	14
	Use of decision support systems and evidence-based policy development	0.0051	19
	Multi-layered financing of the health system to allocate public resources to vulnerable groups	0.005749	11
	Development of resistance economy approach in the health system	0.00342	32
	Integrating basic social insurance funds	0.00934	4
	Outsourcing services with an emphasis on systematic monitoring	0.00448	26
	Strengthening the monitoring of the performance of service delivery units	0.00473	22
	Identifying and reducing induced demand in providing health care based on clinical guidelines	0.00451	25
	Identifying and reducing the moral risks of the insured in the health system	0.00399	28
	Separation of the roles of supervision, provision of care, and monitor the health system	0.00369	29
**Health Sector-Specific Grants and Foreign Aid**	Use of foreign aid	0.00365	30
	Foreign borrowing	0.00239	37
	Foreign aid through technology transfer	0.00356	31
**Conducive Macroeconomic Conditions**	Development of the country’s taxation system	0.00199	41
	Planning to reduce sanctions and the economic effects it caused	0.00084	48
	Increasing the government budget balance	0.00116	47
	Reducing economic dependence on oil exports and underground resources	0.00075	49
	Control and management of the health sector inflation	0.00075	49
**Increase in Health Sector-Specific Resources**	Establishing and increasing earmarked taxes for the health sector	0.01408	1
	Encouraging private investment in the health sector	0.00587	10
	Attracting the participation of non-governmental organizations and philanthropists in the health sector	0.00963	3
	Allocation of resources from the targeting of subsidies to the health sector	0.00687	8
	Modifying and increasing tariffs and insurance premiums	0.00523	18
	Withdrawal from financial funds and strategic reserves of the country in times of health crisis	0.00573	12
	Development of joint healthcare plans and investments with other government agencies	0.004988	20
	donation-based crowdfunding and voluntary participation of people	0.00934	6
	Encouraging foreign investment in health	0.00557	13
	Using the capacity of the capital market for macro healthcare project financing	0.00609	9
	Tax exemptions for economic activists in the health sector during the crisis	0.00930	5
	Allocating a share of public service payments to the health sector	0.00699	7

## Discussion

Over the past 15 years, the health sector has grown faster than the entire economy [14]. This study has identified five strategies and 50 sub-strategies that could serve as a framework for policymakers to develop a tailored model that suits the specific context and needs of their country or region.

### Structure reforms

While the first stage of our study did not identify the strategy of re-prioritizing the health sector within the public budget in the literature on fiscal space in the health sector, this strategy was emphasized as a structural change by the experts in the second stage. This strategy included 11 sub-strategies, with the most common being the re-prioritization of the health budget within the government budget. Three sub-strategies were added by the experts in this stage, with the re-prioritization of the health budget within the government budget being the most important and the development of zero-based budgeting being the least important. In India, the government has adopted decentralization policies to change the budgetary allocation and implementation process to improve the structure (13). A study showed that although Bolivia has a relatively good income, less budget is allocated to the health sector [14].

In addition, the World Health Organization’s report on the re-prioritization of health budget in the government’s general budget suggests sterategies such as improving communication between the Ministry of Health and Finance to strengthen capacity and mutual understanding of the health budget. This has also been addressed in research studies [15, 16]. Furthermore, a study in Indonesia has mentioned that re-prioritization should consider the population age pyramid and the need to respond to future health and medical needs [17].

In our study, sub-strategies falling under this strategy had a lower priority than others since changing the structure and implementation is a challenging task. Moreover, such changes usually take time and are effective in the long term, and are not recommended during a crisis. However, by implementing sub-strategies related to this strategy, access to new potentials that are stable and reliable can be possible, and can be used in future crises.

### Increase in efficiency of health expenditures

Many countries today face resource constraints, including limited productivity, within their fiscal space [18]. Evidence has shown that up to 40% of resources are wasted [14]. Within this strategy, we identified 17 sub-strategies, with the development of the UHC plan having the highest priority according to our analysis. It is worth noting that improving efficiency is a key factor in the effective allocation of limited resources in the health sector. For instance, in India, tackling corruption and money laundering has been identified as one of the most important sub-strategies to improve efficiency [13]. n our study, the most common sub-strategy for improving efficiency was the management of supply and demand, optimal use of available resources, and reduction of resource wastage. Additionally, ensuring the appropriate use of medicine, providing health education for prevention, strategic purchasing, and preventing induced demand were identified as crucial concerns for improving efficiency in the health sector. These findings are consistent with previous studies conducted in Iran and Turkey [12, 19]. Also; in countries in the South American region, the consolidation and integration of healthcare services have been a priority, which is highlighted as a high priority in the conducted research for increasing efficiency.

In South American countries, consolidation and integration of healthcare services and improvement of payment mechanisms have been considered as high priority strategies to increase efficiency [20]. The Italian government has made COVID-19 related testing and treatment costs free/accessible to all people to improve universal health coverage, particularly for the elderly population who are at a higher risk of severe COVID-19 and face poverty challenges UHC [21]. The discussion concludes by noting that implementing sub-strategies related to efficiency can improve access to stable and reliable resources, which can be particularly useful in future crises.

Given Iran’s weakness in the efficiency sector of health, quantifying the effect of efficiency on health expenditures is complex. Given diminishing marginal returns, it is estimated that a relatively small increase in efficiency can create significant fiscal space. Considering the current crisis and regardless of the economic status of a country, increased efficiency is an available and applicable strategy. Fiscal space in the health sector can be created by optimally using the available resources, reducing the waste of resources, and evaluating the efficiency of budgetary allocation. Regarding self-treatment among the Iranian population, it seems the amount of resource wastage can be avoided by training and how to take medicines. Even though the number of people with health insurance has grown in Iran in recent years, 6 to 9 million of the population do not have any health insurance. Therefore, given more than 50% of medical expenses are paid Out-Of-Pocket (OOP), health insurance coverage should be improved.

In Iran, there are three main social health insurance organizations: the Social Security Organization, the Iranian Health Insurance Organization, and the Armed Forces Medical Services Insurance Organization. As of the end of 2019, there were approximately 44 million insured individuals under the Social Security Organization [22], while the Iranian Health Insurance Organization had around 42 million insured individuals. The Rural Insurance Fund, Self-employed Insurance Fund, Government Employees Insurance Fund, and other sectors respectively formed 48%, 13%, 33%, and 6% of the insured population under this organization [23]. However, there are still some people in Iran who do not have health insurance, and some are covered by multiple social health insurances. Despite the recent increase in the number of insured individuals, between 6 and 9 million Iranians are still uninsured. Therefore, it is crucial to remove overlaps and provide insurance services to all members of society, which is an important task for this sector.

### Health sector-specific grants and foreign aid

One way to meet the financial needs of low-income countries under the pressure of foreign loans is financial assistance. Between March and September, the International Monetary Fund (IMF) and multilateral development banks pledged $38 billion to assist developing Asian countries in fighting against COVID-19 [24]. In Libya, financial and humanitarian aid rose considerably during the Ebola outbreak [25]. Many developing countries rely heavily on external aid and assistance during a crisis like COVID-19, and it is expected that low-income countries will continue to depend on developed countries’ aid. However, more investments in the health sector are possible with greater government spending for health [26]. The amount of foreign aid for development in 2018 was about $16 billion (2.0% of the total global health expenditure), which played a significant role in financing health expenditures in low-income countries [27]. Taking out loans as a sub-strategy is another option; however, in Iran, considering the sanctions, the possibility of using this potential is insignificant. Furthermore, the strategy’s unsustainability makes it impossible to rely on it.

### Conducive macroeconomic conditions

Fiscal space for health depends on a conducive macroeconomic environment, such as sustained economic growth, improved income generation, and low levels of fiscal deficit [28]. One of the sources of the government’s revenue is the tax received from the people. Taxes are used for different purposes depending on a country’s circumstances. One of the sectors that tax is allocated to is the health sector. Therefore, receiving more tax and earmarking this as a source of revenue for the health sector can contribute to improvement in the sector.

It has been estimated that during the Covid-19 pandemic, tax administration reforms reap higher tax revenues of about 3–4% of the Gross Domestic Product (GDP) in large economies such as India and Indonesia. The tax base can broaden by rationalizing tax exemptions and introducing new tax instruments. In the Asia-Pacific region, there is also scope for increasing direct taxes such as income tax, property tax, and wealth tax [24]. All of the mentioned items were among the proposed sterategies and were among the prioritized items by the experts.

In this study, five sub-strategies were identified within a conducive macroeconomic conditions strategy. The most important sub-strategy was the development of the country’s tax system. In Peru, direct fossil fuel taxes account for 27% of the country’s total tax revenues. In Peru and Bolivia, governments try to increase their countries’ revenue by increasing direct and indirect taxes [29, 30]. One of the most important pillars to receive taxes is targeting and improving the tax system, which in our study had the highest priority among other sub-strategies falling into this strategy. One of the factors that can lead to a reduction in tax received is tax amnesties, which by structuring them, more revenue is generated [13].

In low-income countries, tax revenue should be 15% of GDP, while in Iran, this figure is less than 6%. This figure is estimated to be 12–17% in neighboring and developed countries and 30–35% in developed countries. In Iran’s sixth development plan, the tax/GDP ratio was set at 10 but was not achieved. It is possible to increase tax revenue by reducing tax amnesties and restructuring tax collection from non-governmental organization jobs and salaried earners. Income tax and wealth tax are highly important as they affect public justice, and evidence has shown that these taxes are associated with health improvement. The next priority is to establish new tax bases. Given the smart plan in line with the implementation of the tax plan in Iran, the percentage of tax received is projected to increase, and the tax gap will decrease [31]. Although this sub-strategy would be effective in the long term, it can be considered a reliable and stable source of income.

### Increase in health sector-specific resources

In our study, 12 sub-strategies were identified within this strategy. The least and most common sub-strategies were imposing and increasing earmarked taxes for the health sector and amending and increasing tariffs and insurance premiums, respectively. Earmarking means taking all or a portion of total revenue from a tax or group of taxes and setting it aside for health [32]. Imposing a tax on goods such as tobacco, cigarettes, and drinks and allocating this tax to the health sector emerged as the most common strategy in eight studies [7, 12, 15, 17, 33–36]. The Indonesian government increased taxes on cigarettes and alcoholic beverages by 4%. In the first year after the reform was implemented, revenue of 23.4 billion pesos and 10.56 billion pesos is expected to be generated from cigarettes and alcoholic beverages, respectively [17]. In Peru, the tobacco tax is estimated to be 37.5%, while high-income countries have a tobacco tax rate of 75%, which accounts for 2% of GDP. In Nigeria, 35% of the excise tax on tobacco revenue is allocated to treatment as part of the budgetary income [3]. Both the Nigerian and Turkish governments are taking steps to encourage private sector investment in the health sector, with Nigeria providing facilities to enhance the capacity of private organizations [37] and Turkey actively encouraging such investment [12].

The government of Ghana financed a portion of its national health insurance costs by increasing value-added tax (VAT) by 5.2% and levying taxes on harmful health products [38, 39]. Similarly, the Gabonese government raised approximately 30 million dollars for the healthcare sector by imposing a 10% tax on two telecommunications companies [40]. All of these examples highlight the importance of increasing taxes to finance the healthcare sector. The most important aspect of increasing taxes is placing them in the right sector and determining the appropriate amount of tax increase. The tax-paying conditions and allocation of taxes to the right sector should be thoroughly examined. Encouraging the private sector to invest in the healthcare sector is one of the sub-sterategies in this category. Given the activities of private organizations in the healthcare sector, providing incentives and encouraging these types of organizations to maximize their potential will improve the overall health conditions of society.

The second most important sub-strategy was related to attracting the participation of NGOs and charitable organizations in the health sector. In Nigeria, the government called on the private sector and local philanthropists to fund the government’s interventions ($72 million) to fight against Covid-19. Religious bodies have also played an important role in the state’s response [37]. In Iran, the average cigarette tax rate is estimated to be 45% in 2022, which is higher compared to high-income countries. This increase serves two purposes: increasing tax revenues that can be allocated directly to health and preventing the consumption of harmful products, thereby reducing future health costs. Providing facilities to private organizations and encouraging them to utilize their maximum capacity can also improve the health status of society.

The sub-strategy of donor giving deserves more attention. More than two thousand NGOs are registered in the Ministry of Health, Treatment, and Medical Education working in the field of health and treatment. During the Covid-19 pandemic, donors donated five billion and 500 million Tomans to the health sector. The proper utilization of this capacity can lead to health funding in different sectors.

### Limitations and strengths

This study comprehensively identifies sterategies and sub-sterategies for developing the fiscal space of the healthcare system in biological crises and prioritizes them. In addition, this study is not solely based on the results of reviewing texts, and researchers have identified hidden variables that were not addressed in previous studies by consulting with experts. Furthermore, the sterategies and sub-sterategies for developing the fiscal space have been categorized through a focused group discussion and prioritized for use in biological crises. Therefore, this study provides a relatively comprehensive model for developing the fiscal space of the healthcare system in crisis situations, so that policymakers and relevant institutions can use it to respond to critical situations. However, like other studies, this study has weaknesses. Limited access to experts was a significant challenge given the research timeframe. Additionally, the number of studies related to fiscal space development in biological crises was limited, and the study could have been improved with a larger number of expert consultations in the second phase.

### Policy implications

Based on the comprehensive model presented in this study for developing the financial sector to finance the cost of treatment in biological crises, health policymakers should consider collaborating with different government departments, such as budget and health organizations, banks, insurance companies, and other institutions. Policymakers are recommended to provide facilities and reduce taxes during biological crises to create the necessary space for the development of private sector activity in the health sector. Additionally, establishing financial funds to cover critical expenses can help provide more sustainable financing during crises. The study acknowledges that financial sector development is only one of the sterategies that can be used to finance treatment costs in biological crises, and it should be accompanied by other financing sterategies. As the study faced limitations in accessing experts and the number of studies related to fiscal space development in biological crises, further research can be conducted to expand the findings and to enhance the implementation of the presented model.

## Conclusion

The current study has provided a comprehensive list of sterategies and sub-sterategies for enhancing preparedness and ensuring sustainable financing in biological crises, based on expert opinions and previous studies. The identified sterategies include structural reforms in the healthcare financing system, increasing special resources for the health sector, improving efficiency, utilizing development aid, and improving macroeconomic conditions. To develop the financial space of the healthcare system, policymakers can consider various approaches such as earmarked health taxes, attracting NGOs and philanthropists, and mobilizing voluntary collective financing. However, it is important to note that the development of the financial space of the healthcare system should not solely focus on increasing financial resources but also emphasize cost-effectiveness and optimal use of resources. In addition, increasing taxes on harmful and luxury goods and allocating them to the health sector is recommended to improve financial resources while expanding universal health coverage schemes during biological crises. Overall, the findings of this study can guide policymakers and relevant institutions in developing effective strategies to enhance preparedness and sustainable financing in biological crises.

## Electronic supplementary material

Below is the link to the electronic supplementary material.

Appendix A

## Data Availability

All data generated or analyzed during this study are included in this published article.
