# Supplementary material for: Providing a model for financing the treatment costs during biological crises using the fiscal space development approach
Source: Health Econ Rev. 2023 Aug 1;13:41. doi: 10.1186/s13561-023-00450-x (PMC10391906; doi:10.1186/s13561-023-00450-x)
Supplement: Supplementary file 1 — Appendix A [file 13561_2023_450_MOESM1_ESM.docx]

**Table 1.** **Search terms used in Scopus, PubMed, and Google Scholar for the systematic review**

| **Search engine** | **Search terms** | **Findings (N)** |
| --- | --- | --- |
| **Scopus** | TITLE(health) AND TITLE("fiscal Space") OR TITLE(financing) OR TITLE(Resource mobilization) OR TITLE(budget) OR TITLE("Earmarking Revenue") AND (TITLE(Epidemic) OR TITLE(Pandemic) OR TITLE(Crisis)) | 32 |
| **Google Scholar** | allintitle: health Financing OR Budget "fiscal space." | 19 |
| **PubMed** | (("Fiscal space"[Title] OR "finance"[Title] OR "budget"[Title] OR "resource mobilization"[Title])) AND ("Health"[Title] ) AND ("crisis"[Title] OR "pandemic"[Title]) | 9 |
